# Frequent copy number variations of PI3K/AKT pathway and aberrant protein expressions of PI3K subunits are associated with inferior survival in diffuse large B cell lymphoma

**DOI:** 10.1186/1479-5876-12-10

**Published:** 2014-01-13

**Authors:** Wenli Cui, Ying Cai, Weige Wang, Zebing Liu, Ping Wei, Rui Bi, Weixiang Chen, Menghong Sun, Xiaoyan Zhou

**Affiliations:** 1 Department of Pathology, Fudan University Shanghai Cancer Center, 270 Dong’an Road, Shanghai 200032, PR China; 2 Department of Oncology, Shanghai Medical College, Fudan University, Shanghai 200032, PR China; 3 Institute of Pathology, Fudan University, Shanghai 200032, PR China; 4 Department of Pathology, The First Affiliated Hospital of Xinjiang Medical University, Urumqi 830054, Xinjiang Uygur Autonomous Region, PR China

**Keywords:** DLBCL, CNV, PI3K/AKT, Subunits, Survival

## Abstract

**Background:**

It has been reported that the PI3K/AKT signaling pathway is activated in diffuse large B-cell lymphoma (DLBCL), PI3K constitutive activation plays a crucial role in PI3K/AKT pathway. However, the copy number variations (CNVs) of PI3K subunits on gene level remain unknown in DLBCL. Therefore, the aim of the study is to investigate the CNV of PI3K subunits and their relationship with clinicopathological features exploring the possible mechanism underlying of PI3K activation in DLBCL.

**Methods:**

CNV of 12 genes in the PI3K/AKT pathway was detected by NanoString nCounter in 60 *de novo* DLBCLs and 10 reactive hyperplasia specimens as controls. Meanwhile, immunohistochemistry (IHC) was performed to examine the expression of p110α, p110β, p110γ, p110δ, and pAKT on DLBCL tissue microarrays.

**Results:**

All PI3K and AKT subunits, except for PIK3R1, had various CNVs in the form of copy number amplifications and copy number losses. Their rates were in the range of 8.3–20.0%. Of them PIK3CA and PIK3CB gene CNVs were significantly associated with decreased overall survival (*P* = 0.029 and *P* = 0.019, respectively). IHC showed that the frequency of strong positive expression of p110α, p110β, p110γ, and p110δ were 26.7%, 25.0%, 18.3%, and 25.0% respectively, and they were found to be associated with decreased survival (*P* = 0.022, *P* = 0.015, *P* = 0.015, and *P* = 0.008, respectively). Expression of p110α was not only significantly associated with CNVs of PIK3CA (*P* = 0.002) but also positively correlated with strong positive expression of pAKT (*P* = 0.026).

**Conclusions:**

CNV of PIK3CA is highly associated with aberrant p110α protein expression and subsequent activation of PI3K/AKT pathway. CNVs of PIK3CA and PIK3CB, and aberrant protein expression of p110 isoforms are of great important value for predicting inferior prognosis in DLBCL. Frequent CNVs of PI3K/AKT subunits may play an important role in the tumorigenesis of DLBCL.

## Background

Diffuse large B-cell lymphoma (DLBCL) is the most common non-Hodgkin’s lymphoma, accounting for 30–40% of adult non-Hodgkin’s malignant lymphoma [1]. Although patients diagnosed with DLBCL are potentially curable with chemotherapy, the disease proves to be fatal in approximately 50% of patients [2]. Recently, given that there has been an increasing trend in the incidence of DLBCL, it is imperative to develop specific and effective treatments related to the pathogenesis of the disease.

Previous studies have reported that the phosphatidylinositol 3-kinase (PI3K) signaling pathway plays a critical role in regulating the growth and survival of DLBCL cells [3], and that constitutive phosphorylation of PI3K resulted in the activation of signaling that represented frequent events both for main pathway components and their downstream substrates [4]. Activated PI3K/AKT signaling pathways have been reported to be associated with decreased disease-free survival (DFS) and a poor response to treatment in patients with DLBCL [5]. This suggests that the PI3K/AKT pathway is potentially an important tumorigenic signaling route and an unfavorable prognostic factor in DLBCL.

PI3Ks consist of a large and complex family that contains three classes, I, II, and III. Of them, Class I PI3K is the most studied and plays a key role in the development and progression of tumors [6]. Class I contains the class IA catalytic subunits PIK3CA, PIK3CB, PIK3CD, and class IB catalytic subunit PIK3CG and the regulatory subunits PIK3R1, PIK3R2, and PIK3R3, while class II contains the catalytic subunits PIK3C2A, PIK3C2B, and PIK3C2G [4]. However, how each subunit precisely contributes to the progression and maintenance of tumors is largely undetermined. The PI3K/AKT signaling pathway can be activated by two main mechanisms: activating mutations and amplifications [7]. Amplification of genes encoding the catalytic subunits of PIK3CA, PIK3CB, PIK3CD, and PIK3CG has been reported in numerous solid tumors [7]. In lymphomas, PIK3CA has been reported to be amplified in 15/22 (68%) cases of mantle cell lymphoma (MCL) [8], 9/161 (5.6%) cases of chronic lymphocytic leukemia (CLL) [9], and mutated in 1/76 (1.3%) cases of DLBCL [10]; while PIK3CD has been reported to be mutated in 3/73 (4.1%) cases of DLBCL [11]. However, there have been few reports available regarding CNVs or mutations of other PI3K/AKT subunits and their contribution to the activation of the PI3K/AKT pathway in DLBCL.

In the present study, we focused mainly on the various PI3K/AKT subunits and profiled their CNVs using the NanoString nCounter assay and investigated their protein expression by immunohistochemistry (IHC). Furthermore, we analyzed the association of CNVs and protein expression with clinicopathological parameters in DLBCL. We also studied various members of the PI3K/AKT pathway simultaneously in the same set of DLBCL clinical samples as well as in a panel of lymphoma cell lines to investigate their involvement in the pathogenesis of DLBCL.

## Materials and methods

### Tissue specimens, cell lines, and patient information

A total of 70 fresh frozen samples including 60 DLBCLs and 10 lymph node reactive hyperplasias (RHs) were collected for CNV detection on the NanoString nCounter platform (NanoString Technologies, Seattle, WA, USA). DLBCL cell lines (DOHH2, OCI-Ly1, OCI-Ly8, and Toledo) and Burkitt’s lymphoma (BL) cell lines (Raji, Namalwa) were included. Corresponding DLBCL formalin-fixed, paraffin-embedded (FFPE) tissues were collected for IHC detection of PI3K catalytic subunits. All cases used in the present study were retrieved during an 8-year period (2005–2012) from our tumor bank and the Department of Pathology in the Shanghai Cancer Center. Diagnoses were reviewed by two pathologists (Wenli Cui and Ying Cai) based on the 2010 World Health Organization classification. The other relevant clinical pathological information including primary site, B symptoms, bulky disease, performance status, lactate dehydrogenase (LDH) activity, stage, International Prognostic Index (IPI) were collected. The DLBCLs were classified into GCB and non-GCB subtype according to Hans algorithm [12]. 13/60 (22%) patients received R-CHOP (Rituximab, cyclophosphamide, doxorubicin, vincristine, and prednisone) or R-CHOP-like therapy, and 47/60 (78%) patients received CHOP or CHOP-like therapy. Patient clinical information was extracted from hospital records. Research protocols for this study were approved by the Ethics Committee at Fudan University Shanghai Cancer Center (Shanghai, China).

### NanoString nCounter assay

Genomic DNA from fresh frozen tissue and cultured cell lines was extracted using a DNA extraction kit (Qiagen, Venlo, Netherlands) following the manufacturer’s protocol. Only DNA samples with an OD A260/280 ratio between 1.7 and 1.9, which indicates optimal purity for DNA, were used for further study. For detection of CNVs in the PI3K/AKT pathway, a panel of custom-compiled gene probes related to the pathway, including PI3K catalytic subunits PIK3CA, PIK3CB, PIK3CD, PIK3CG, PIK3C2A, PIK3C2B, and PIK3C2G, and regulated subunits PIK3R1 and PIK3R2, as well as AKT subunits AKT1, AKT2, and AKT3 were designed using NanoString nCounter technology and subsequently analyzed on the NanoString nCounter platform. NanoString probes were designed for the 12 genes according to different exons located in different regions (Table 1). Three probes were designed for each gene. Each assay contained six positive dsDNA control probes, eight negative control probes, and 10 invariant control probes (INVs) designed for autosomal genomic regions predicted not to contain common CNVs.

**Table 1:** PI3K/AKT subunits, their localization and exons for probes

**Genes**	**Localization**	**Exons for probes**
PIK3CA	3q26.3	9,10,20
PIK3CB	3q22.3	8,11,22
PIK3CD	1p36.2	1,4,10
PIK3CG	7q22.3	2,9,10
PIK3R1	5q13.1	8,13,15
PIK3R2	19q13.2-q13.4	2,8,15
PIK3C2A	11p15.5-p14	1,10,32
PIK3C2B	1q32	3,14,35
PIK3C2G	12p12	2,16,26
AKT1	14q32.32	2,4,13
AKT2	19q13.2	4,5,11
AKT3	1q44	4,7,12

The NanoString nCounter assay was performed according to NanoString’s standard protocol. Briefly, 600 ng of fragmented genomic DNA per assay was hybridized with the capture and reporter probes in 30 μL total volume and incubated overnight at 65°C for at least 16 h. The target and probe complexes were washed and immobilized in the cartridge. Genomic DNA was fragmented into small pieces (200–800 bp) and denatured to produce single strands. The custom CNV CodeSet was then hybridized to the fragmented denatured DNA sample in a single multiplexed reaction (up to 800 genomic loci per CodeSet). Hybridized DNA-CodeSet complexes were purified using the fully automated nCounter prep station, and reporters were counted using the nCounter digital analyzer. The data were normalized to the INVs and to positive and negative controls in each hybridization reaction. Finally, data analysis was performed using nSolver software.

Copy number was determined by averaging over three probes per region. If the average copy number was below 1.4, the gene was considered as one copy; if between 1.5 and 2.4, considered as two copies; and if between 2.5 and 3.4, considered as three copies, according to the manufacturer’s protocol.

### Immunohistochemistry

IHC was performed to detect expression of the four PI3K catalytic isoforms and pAKT on routinely processed FFPE specimens from which tissue microarrays (TMAs) were constructed using double cores (core diameter was 1 mm) from selected areas of 60 cases of DLBCL and 10 cases of RH. Briefly, serial sections from FFPE samples were collected onto poly-L-lysine coated slides and processed with a standard manual streptavidin-peroxidase technique using a biotin-free detection system (Dako, Carpinteria,CA, USA) after a heat-induced antigen retrieval procedure with EDTA for pAKT and citric acid for other antigens for 3 min. Primary antibodies were incubated with TMAs overnight. Omission of the primary antibody and its replacement with an antibody diluent were used as the negative control. A ready-to-use kit (EnVision™; Dako) was used according to the manufacturer’s instructions.

The source and dilution of the primary antibodies used were as follows: PI3K p110α (#4249, clone C73F8, 1:400), PI3K p110β (#3011, clone C33D4, 1:400), and PI3K p110γ (#5405, clone D55D5, 1:200) were from Cell Signaling Technology (Beverly, MA, USA), PI3K p110δ (sc-7176, clone H-219, 1:200) was from Santa Cruz Biotechnology (Dallas, TX, USA), and pAKT (Ser 473) (#2118-1, clone EP2109Y, 1:150) was from Epitomics (Burlingame, CA, USA).

### Scoring of immunostaining

Expression of the four PI3K catalytic isoforms was evaluated blindly and independently by two pathologists (Wenli Cui and Ying Cai). The staining intensity was scored from 0 to 3: 0, no appreciable staining in tumor cells (negative); 1, slight staining in tumor cells (weak positive); 2: moderate staining in tumor cells (moderate positive); 3: distinct staining in tumor cells (strong positive) [5]. Cases were considered positive if ≥30% of the tumor cells were stained with an antibody, as described in a previous study involving IHC evaluation of TMAs [12].

### Statistical analysis

Overall survival (OS) was measured from date of diagnosis to date of death of any cause, or latest follow-up. Survival analysis was performed using Kaplan–Meier survival curves with a statistical software package (IBM SPSS Statistics 20.0, Armonk, Town of North Castle, NY, USA) and compared with the use of logistic statistics (log-rank). The Fisher’s exact or χ^2^ test was used for statistical analysis of categorical data. The Cox proportional hazard regression model was used for univariate analyses. A *P*-value <0.05 was considered statistically significant.

## Results

### CNV of 12 genes in the PI3K/AKT pathway in DLBCL

We analyzed CNVs of 12 genes in a total of 60 DLBCLs, six cell lines including DOHH2, OCI-Ly1, OCI-Ly8, Toledo, Raji, and Namalwa, and 10 lymphoid hyperplasias. The results showed that 11 of the 12 PI3K/AKT family members had significant CNVs, including copy number amplifications and copy number losses. These were PIK3CA (17%), PIK3CB (20%), PIK3CD (17%), PIK3CG (8%), PIK3C2A (8%), PIK3C2B (13%), PIK3C2G (20%), PIK3R2 (23%), AKT1 (18%), AKT2 (18%), and AKT3 (22%) (Table 2). Among genes with CNVs, we found that the copy numbers of PIK3CA, PIK3CB, PIK3R2, and PIK3C2B were only amplified (10/10, 12/12, 14/14, and 8/8, respectively); copy numbers of PIK3CG, PIK3C2A, PIK3C2G, AKT1, AKT2, and AKT3 were amplified in the majority of cases (4/5, 4/5, 11/12, 7/11, 8/11, and 11/13, respectively), whereas copy number losses occurred in fewer cases (1/5, 1/5, 1/12, 4/11, 3/11, and 2/13, respectively); copy number losses for PIK3CD occurred in the majority of cases (7/10), whereas amplifications occurred in a few cases (3/10) (Table 3). In DLBCL, the CNV frequency of PI3K-AKT subunits was in the range of 1–10 genes; in one of the 60 DLBCLs, 10 different genes were detected with CNVs. In this sample set, 58.3% (35/60) of patients had at least one CNV and 48.3% (29/60) had at least two co-occurring CNVs in the component of PI3K/AKT subunit genes (Table 4). CNVs were identified in most of the PI3K/AKT family genes in DLBCL. There was no statistically correlation found between any CNVs of PI3K gene family and that of AKT gene family, indicating there was no cause-and-effect interplay between CNVs of PI3K and AKT.

**Table 2:** The frequency CNV of PI3K/AKT subunits in 60 DLBCLs by NanoString nCounter analysis

**Gene**	**Case no.**	**Copy number amplification no. (%)**	**Copy number loss no. (%)**	**Total CNV frequency no. (%)**
PIK3CA	60	10 (17)	0 (0)	10 (17)
PIK3CB	60	12 (20)	0 (0)	12 (20)
PIK3CD	60	3 (5)	7 (12)	10 (17)
PIK3CG	60	4 (6)	1 (2)	5 (8)
PIK3R1	60	0 (0)	0 (0)	0 (0)
PIK3R2	60	14 (23)	0 (0)	14 (23)
PIK3C2A	60	4 (6)	1 (2)	5 (8)
PIK3C2B	60	8 (13)	0 (0)	8 (13)
PIK3C2G	60	11 (18)	1 (2)	12 (20)
AKT1	60	7 (12)	4 (6)	11 (18)
AKT2	60	8 (13)	3 (5)	11 (18)
AKT3	60	11 (18)	2 (3)	13 (22)

**Table 3:** The distribution of varied CNV in each PI3K/AKT subunits by NanoString nCounter analysis

**Gene**	**Total CNV no.**	**Copy number loss no. (%)**	**Copy number amplification no. (%)**			**Total copy number amplification no. (%)**
		**1 copy**	**3 copy**	**4 copy**	**6 copy**	
PIK3CA	10	0 (0)	9 (90)	1 (10)	0 (0)	10 (100)
PIK3CB	12	0 (0)	11 (92)	1 (8)	0 (0)	12 (100)
PIK3CD	10	7 (70)	2 (20)	1 (10)	0 (0)	0 (30)
PIK3CG	5	1 (20)	4 (80)	0 (0)	0 (0)	4 (80)
PIK3R1	0	0 (0)	0 (0)	0 (0)	0 (0)	0 (0)
PIK3R2	14	0 (0)	12 (86)	1 (7)	1 (7)	14 (100)
PIK3C2A	5	1 (20)	4 (80)	0 (0)	0 (0)	4 (80)
PIK3C2B	8	0 (0)	8 (100)	0 (0)	0 (0)	8 (100)
PIK3C2G	12	1 (8)	10 (84)	1 (8)	0 (0)	11 (92)
AKT1	11	4 (36)	6 (55)	1 (9)	0 (0)	7 (64)
AKT2	11	3 (27)	8 (73)	0 (0)	0 (0)	8 (73)
AKT3	13	2 (15)	9 (69)	2 (16)	0 (0)	11 (85)

**Table 4:** CNV of PI3K/AKT subunits genes involved in 60 DLBCLs

	**Genes involoved (≥)**	**Total case no.**	**CNV no.**	**Frequency**
PI3K/AKT subunits	1 genes	60	35	58%
	2 genes	60	29	48%
	3 genes	60	25	42%
	4 genes	60	18	30%
	5 genes	60	12	20%
	6 genes	60	6	10%
	7 genes	60	3	5%
	9 genes	60	2	3%
	10 genes	60	1	2%

PIK3CG was found to be amplified in all GCB cell lines, including DOHH2, OCI-Ly1, OCI-Ly8, and Toledo; PIK3C2A and AKT1 were amplified in OCI-Ly1, OCI-Ly8, and Toledo; while PIK3C2B and PIK3C2G were amplified only in Toledo (Table 5).

**Table 5:** Copy number of PI3K/AKT pathway in DLBCLs and BLs cell lines

	**DLBCL**				**BL**	
	**DOHH2**	**OCI-LY1**	**OCI-LY8**	**Toledo**	**Raji**	**Namalwa**
PIK3CA	2	2	2	2	2	2
PIK3CB	2	2	2	2	2	2
PIK3CD	2	2	2	2	2	2
PIK3CG	3	3	3	3	2	2
PIK3R1	2	2	2	2	2	2
PIK3R2	2	2	2	2	2	2
PIK3C2A	2	3	3	3	2	2
PIK3C2B	2	2	2	3	2	2
PIK3C2G	2	2	2	3	2	2
AKT1	2	3	3	3	2	2
AKT2	2	2	2	2	2	2
AKT3	2	2	2	4	2	2

### Protein expression of PI3K catalytic subunits in DLBCL

**Figure 1:**
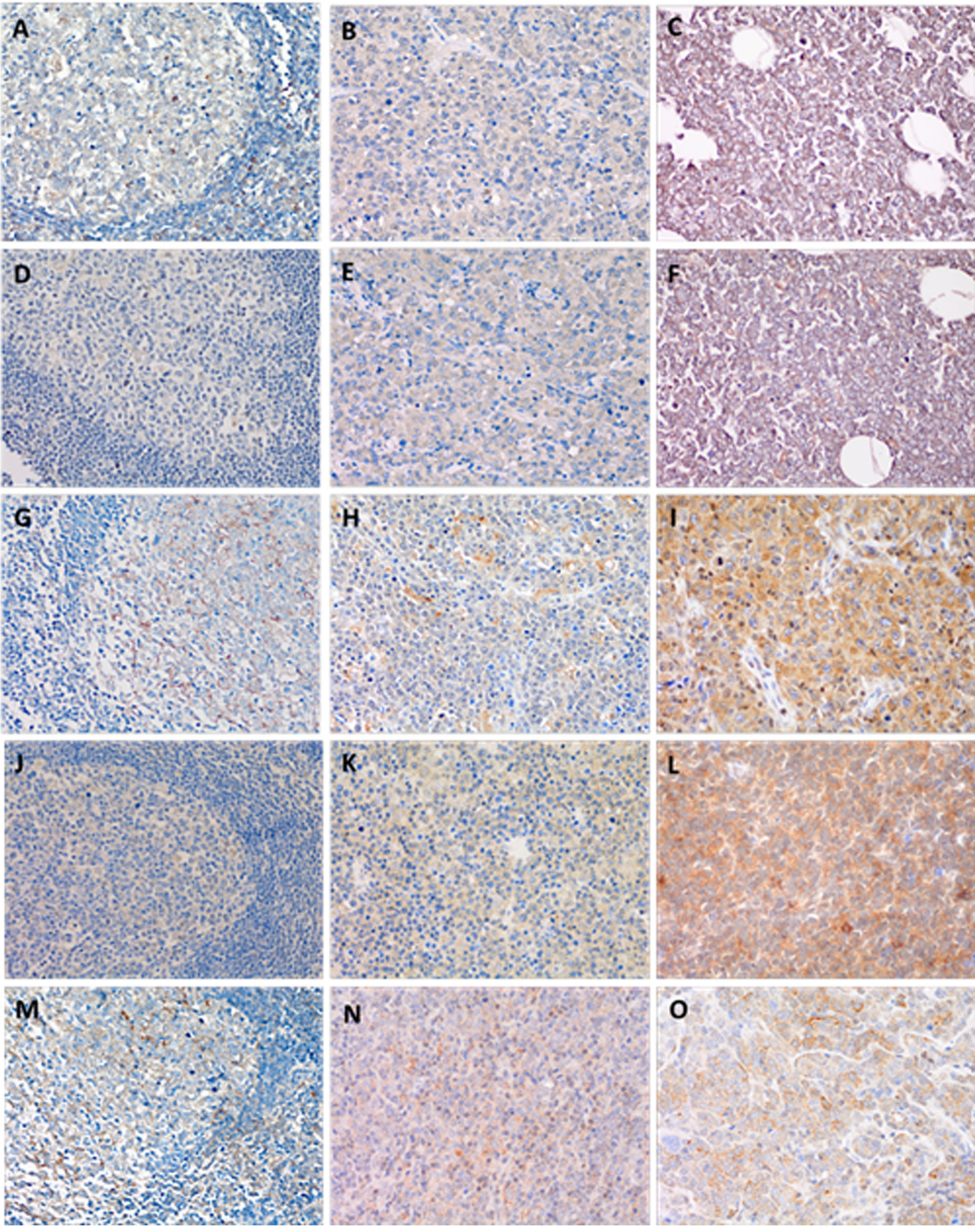
**Protein expression of p110α, p110β, p110γ, p110δ, and pAKT was performed on tissue microarrays by Immunohistochemistry (IHC). A-C**. Weak positive of germinal center in RH, weak positive and strong positive of p110α in DLBCL **D-F**. Weak positive of germinal center in RH, weak positive and strong positive of p110β in DLBCL **G-I**. Weak positive of germinal center in RH, weak positive and strong positive of p110γ in DLBCL **J-L**. Weak positive of germinal center in RH, weak positive and strong positive of p110δ in DLBCL **M-O**. Weak positive of germinal center in RH, weak positive and strong positive of pAKT in DLBCL.

To further investigate the association between CNVs of PI3K subunits and their protein expression, and the significance of Class I subunits in DLBCL, we analyzed the expression of p110α, p110β, p110γ, p110δ, and pAKT protein in a TMA of 60 DLBCLs using IHC. All of these proteins were expressed in the cytoplasm. In DLBCL, proteins were diffusely expressed in tumor cells, while in RH they were locally expressed in germinal centers (Figure 1). The expression frequencies of p110α, p110β, p110γ, p110δ, and pAKT protein were 80%, 81.6%, 81.6%, 81.6%, and 75%, respectively. Strong positive expression of the above proteins was found in 26.7%, 25.0%, 25.0%, 18.3%, and 16.7% of cases, respectively. Among the four PI3K subunit proteins expressed, only p110α showed strong positive expression, which was positively correlated with CNVs of PIK3CA (*P* = 0.002). P110α strong positive expression was also correlated with strong positive expression of pAKT (*P* = 0.026). Other strong positive expressions of p110β, p110γ, and p110δ have no correlation with CNVs of PIK3CB, PIK3CG and PIK3CD. There was no significant correlation between the expression of these p110 isoforms and expression of pAKT.

### Association between CNVs in PI3K/AKT genes and clinicopathological characteristics in DLBCL

**Figure 2:**
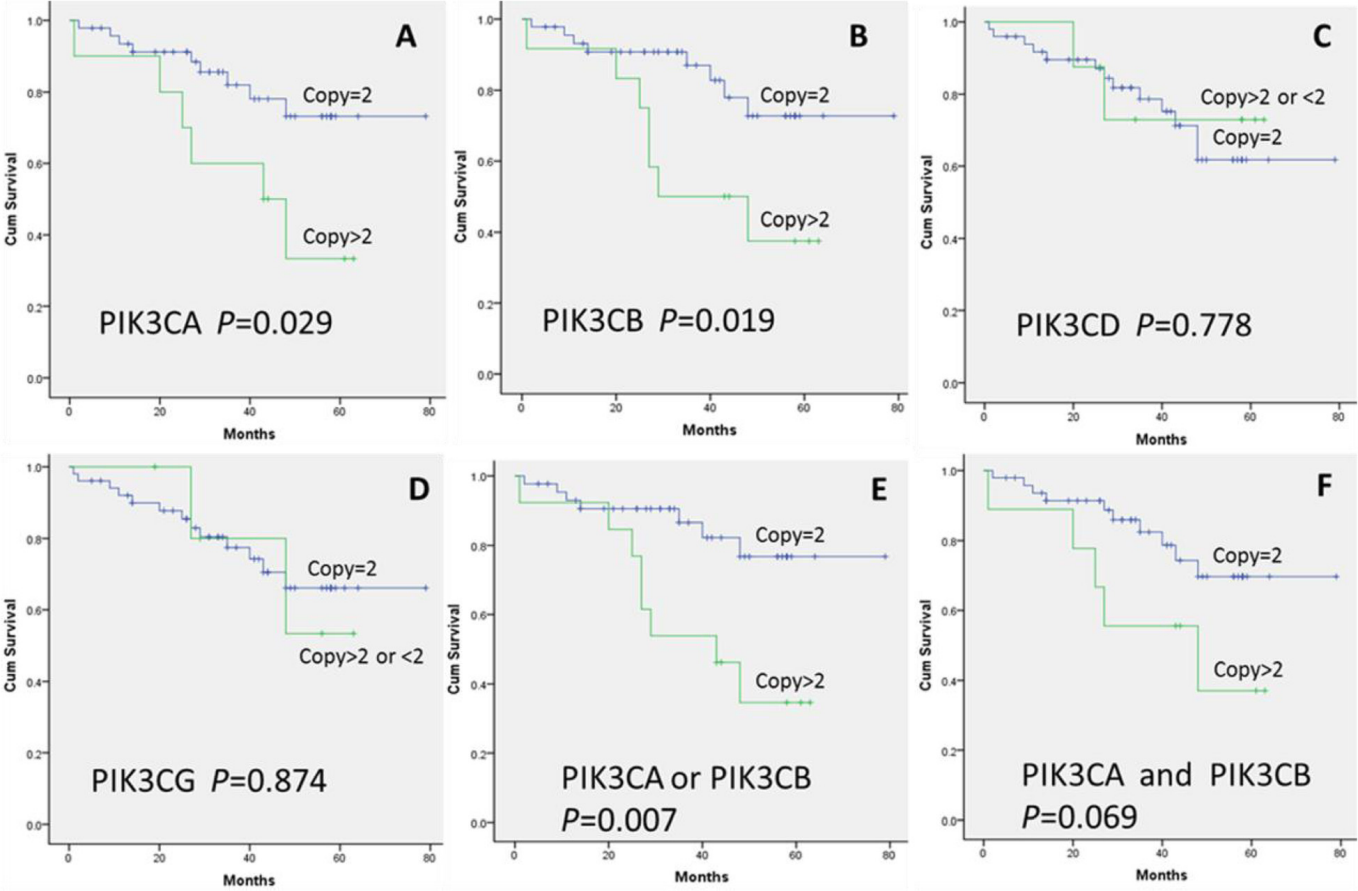
**Correlation of CNVs of PIK3CA, PIK3CB, PIK3CD, PIK3CG by NanoString nCounter analysis with DLBCL survival.** It showed that CNV of PIK3CA, PIK3CB were associated with decreased survival. Other PI3K/AKT subunits represented no correlation. **A**. Patients grouped by PIK3CA CNV(*P* = 0.029). **B**. Patients grouped by PIK3CB CNV(*P* =0.019). **C**. Patients grouped by PIK3CD CNV(*P* =0.778). **D**. Patients grouped by PIK3CG CNV(*P* =0.874). **E**. Patients grouped by either PIKCA or PIK3CB CNV(*P* =0.007). **F**. Patients grouped by both PIKCA and PIK3CB CNV(*P* =0.069).

Among the 60 patients with DLBCLs, their ages were in the range of 21–86 years with a mean age of 58 years. Fifty-seven cases had follow-up data from 2 to 79 months, with the average period being 34 months. During this period, 15/57 (26%) patients died. There was a significant association of shorter survival with CNVs of PIK3CA and PIK3CB (Figure 2). Patients with CNVs of PIK3CA and PIK3CB had significantly shorter survival times respectively (*P* = 0.029, n = 10; *P* = 0.019, n = 12) than those with two wild-type copies (Figure 2A, Figure 2B). Patients whose DLBCLs had either PIK3CA or PIK3CB CNVs had significantly shorter survival times (*P* = 0.007, n = 13) than those without CNVs (Figure 2E). Both PIK3CA and PIK3CB CNVs had no significant shorter survival times (*P* = 0.069, n = 8) than those without CNVs (Figure 2F). No significant differences or similarities in survival were seen for patients with CNVs of PIK3CD (Figure 2C), PIK3CG (Figure 2D), PIK3C2A, PIK3C2B, PIK3C2G, PIK3R2, AKT1, AKT2, or AKT3. CNVs of PIK3CA and PIK3CB were higher in the non-GCB DLBCLs (n = 8 and n = 10, respectively) than in the GCB DLBCLs (n = 2 and n = 2, respectively). No difference in different pathological types was seen in other subunits. There were no significant differences between CNVs of PIK3CA, PIK3CB, PIK3CD, and PIK3CG with clinicopathological characteristics, including sex, age, primary site, B symptoms, bulky disease, performance status, LDH activity, stage, IPI, or pathological type (Table 6). Clinicopathological characteristics had no impact on survival through Cox regression univariate analysis.

**Table 6:** CNV of PI3K subunits in 60 patients with DLBCLs by Nanostring nCounter analysis

		**PIK3CA**	***P *value**	**PIK3CB**	***P *value**	**PIK3CD**	***P *value**	**PIK3CG**	***P *value**
Total no. of patients	60								
Sex									
Male	39	5 (12.8)	0.298	7 (17.9)	0.737	10 (25.6)	0.011	5 (12.8)	0.412
Female	21	5 (23.8)		5 (23.8)		0 (0)		1 (4.8)	
Age, years									
<60 y	36	6 (16.7)	1.000	5 (13.9)	0.193	6 (16.7)	1.000	5 (13.9)	0.387
≥60 y	24	4 (16.7)		7 (29.2)		4 (16.7)		1 (4.2)	
Primary site									
node	54	10 (18.5)	0.557	11 (20.4)	1.000	9 (16.7)	1.000	6 (11.1)	1.000
Perpheral node	6	0 (0)		1 (16.7)		1 (16.7)		0 (0.0)	
B symptoms									
Absence	37	7 (18.9)	0.727	7 (18.9)	1.000	6 (16.2)	1.000	2 (5.4)	0.191
Presence	23	3 (13.0)		5 (21.7)		4 (17.4)		4 (17.4)	
Bulky disease									
<10 cm	47	7 (14.9)	0.506	8 (17.0)	0.552	7 (14.9)	1.000	4 (8.5)	0.347
≥10 cm	4	1 (25.0)		1 (25.0)		0 (0)		1 (25.0)	
Performance status									
ECOG 0-1	50	8 (16.0)	1.000	8 (16.0)	1.000	7 (14.0)	0.125	6 (12.0)	1.000
ECOG 2-4	4	0 (0.0)		1 (25.0)		2 (50.0)		0 (0)	
LDH									
<2*insitutioal ULN	30	6 (34.9)	0.715	6 (20.0)	1.000	6 (20.0)	0.117	3 (10.0)	1.000
>2*insitutioal ULN	24	3 (12.5)		5 (20.8)		1 (4.2)		3 (12.5)	
Extranodal site									
≤1	50	9 (18.0)	1.000	11 (22.0)	0.571	7 (14.0)	1.000	6 (12.0)	1.000
>1	4	0 (0)		0 (0.0)		0 (0)		0/4 (0)	
Stage									
I/II	29	3 (10.3)	0.161	3 (10.3)	0.087	4 (13.8)	1.000	1 (3.4)	0.076
III/IV	23	6 (26.1)		7 (30.4)		3 (13.0)		5 (21.7)	
IPI									
0-1	30	5 (16.7)	1.000	4 (13.3)	0.198	4 (13.3)	1.000	4 (13.3)	0.678
2-5	25	4 (16.0)		7 (28.0)		4 (16.0)		2 (8.0)	
Pathology									
GCB	26	2 (76.9)	0.163	3 (11.5)	0.152	5 (19.2)	0.733	3 (11.5)	1.000
non-GCB	34	8 (23.5)		9 (26.5)		5 (14.7)		3 (8.8)	

*DLBCL* diffuse large B-cell lymphoma, *GCB* germinal center B cell, *IPI* international prognostic index, *LDH* lactate dehydrogenase.

### Association between protein expression of PI3K catalytic subunits and clinicopathological features of DLBCL

**Figure 3:**
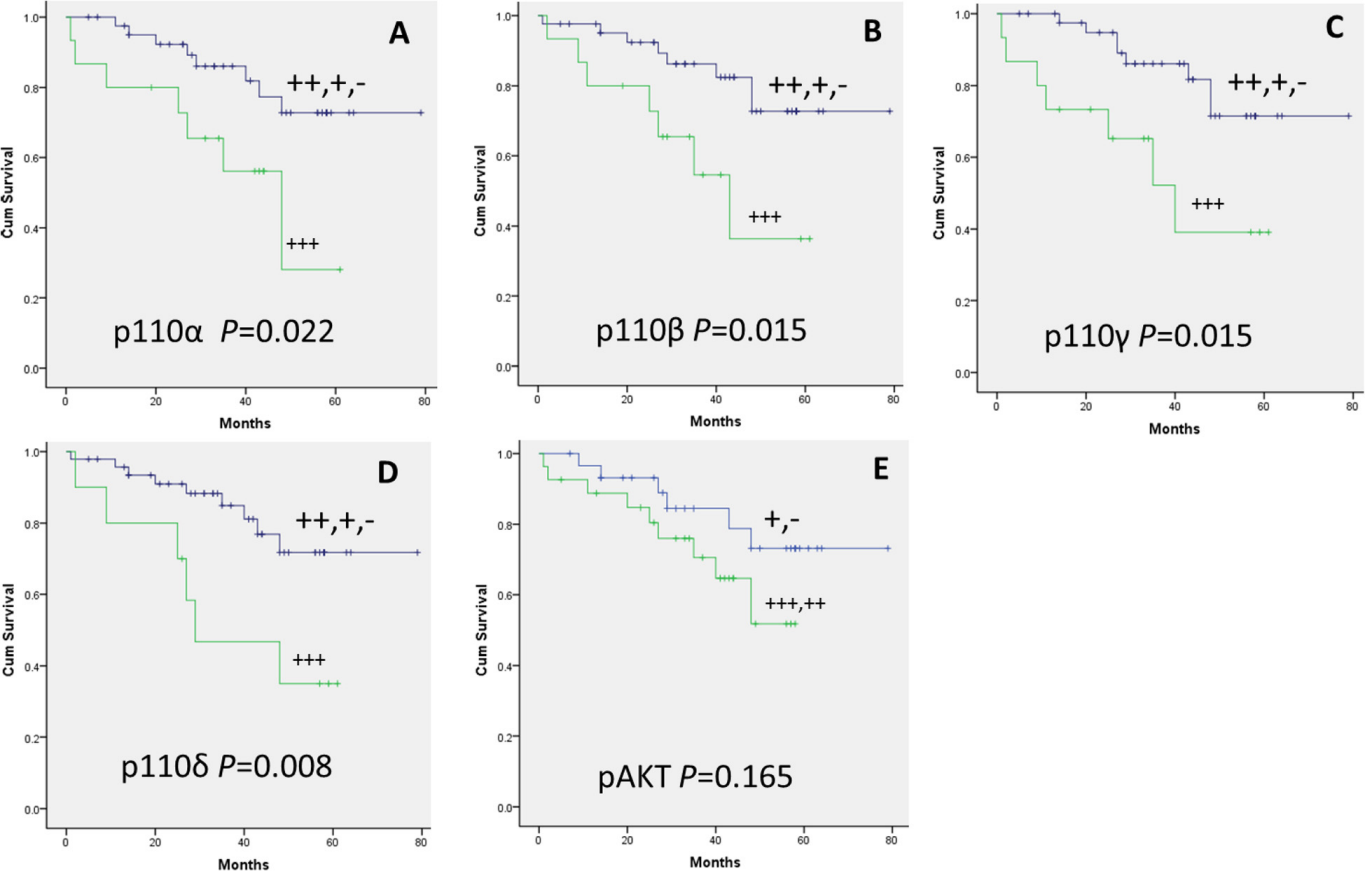
**Protein expression of p110α, p110β, p110γ, p110δ, and pAKT correlated with patient survival in DLBCL.** It showed that strong positive expression of p110α, p110β, p110γ, and p110δ were associated with decreased survival. Moderate and strong positive expression of pAKT showed correlation with decreased survive, however, there is no statistical significance. **A**. Patients grouped by strong positive expression of p110α(*P* = 0.022). **B**. Patients grouped by strong positive expression of p110β(*P* =0.015). **C**. Patients grouped by strong positive expression of p110γ(*P* =0.015). **D**. Patients grouped by strong positive expression of p110δ(*P* =0.008). **E**. Patients grouped by moderate and strong positive of pAKT(*P* =0.165).

There were no positive correlations between strong positive expression of p110α, p110β, p110γ, and p110δ with clinicopathological characteristics, including sex, age, primary site, B symptoms, bulky disease, performance status, LDH activity, stage, IPI, and pathological type, except for p110δ, which had a significant difference in high IPI (*P* = 0.037) (Table 7). Strong positive expression of p110α, p110β, p110γ, and p110δ was found to be associated with decreased survival (*P* = 0.022, *P* = 0.015, *P* = 0.015, and *P* = 0.008 respectively) (Figure 3A, 3B, 3C, 3D). Strong and moderate expression of pAKT associated with decreased survival (*P* = 0.165) (Figure 3E).

**Table 7:** Strong positive expresssion of p110 isforms and pAKT in 60 patients with DLBCLs by IHC

		**p110α**	***P *value**	**p110β**	***P *value**	**p110δ**	***P *value**	**p110γ**	***P *value**	**pAKT**	***P *value**
Total no. of patients	60										
Age, years											
<60 y	36	12 (33.3)	**0.037**	10 (27.8)	0.639	12 (33.3)	0.068	7 (19.4)	1.000	7 (19)	0.293
≥60 y	24	4 (16.7)		5 (20.8)		3 (12.5)		4 (16.7)		2 (8.3)	
Sex											
Male	39	7 (17.9)	0.153	9 (23.1)	0.762	11 (28.2)	0.435	7 (17.9)	1.000	7 (17.9)	0.473
Female	21	9 (42.9)		6 (28.6)		4 (19.0)		4 (19.0)		2 (9.5)	
Primary site											
node	54	14 (25.9)	0.653	14 (25.9)	1.000	14 (25.9)	1.000	8 (14.8)	0.069	8 (14.8)	1.000
Perpheral node	6	2 (33.3)		1 (26.7)		1 (16.7)		3 (50.0)		1 (16.6)	
B symptoms											
Absence	37	10 (27.0)	0.936	10 (27.0)	0.764	11 (29.7)	0.283	6 (16.2)	0.734	4 (10.8)	0.284
Presence	23	6 (26.1)		5 (21.7)		4 (17.4)		5 (21.7)		5 (21.7)	
Bulky disease											
<10 cm	47	11 (23.4)	1.000	13 (27.7)	0.561	12 (25.5)	1.000	8 (17.0)	0.552	7 (14.9)	1.000
≥10 cm	4	1 (25.0)		1 (0)		1 (25.0)		1 (25.0)		0 (0)	
Performance status											
ECOG 0-1	50	13 (26.0)	1.000	13 (26.0)	0.965	12 (24.0)	1.000	9 (18.0)	0.571	8 (16)	1.000
ECOG 2-4	4	1 (25.0)		1 (25.0)		1 (25.0)		1 (25.0)		0 (0)	
LDH											
<2*insitutioal ULN	30	9 (30.0)	0.445	11 (36.7)	0.133	9 (30.0)	0.445	7 (23.3)	0.483	6 (20)	0.277
>2*insitutioal ULN	24	5 (20.8)		4 (16.7)		5 (20.8)		3 (12.5)		2 (8.3)	
Extranodal site											
≤1	50	13 (26.0)	1.000	14 (28.0)	1.000	13 (26.0)	1.000	9 (18.0)	0.571	8 (16)	1.000
>1	4	1 (25.0)		1 (25.0)		1 (25.0)		1 (25.0)		0 (0)	
Stage											
I/II	29	5 (17.2)	0.077	8 (27.6)	0.904	10 (34.5)	0.168	4 (13.8)	0.307	4 (13.8)	1.000
III/IV	23	9 (39.1)		6 (26.1)		4 (17.4)		6 (26.1)		4 (17.4)	
IPI											
0-1	30	10 (33.3)	0.142	11 (36.7)	0.087	11 (36.7)	**0.037**	5 (16.7)	1.000	6 (20)	0.269
2-5	25	4 (16.0)		4 (16.0)		3 (12.0)		5 (20.0)		2 (8)	
Pathological type											
GCB	26	4 (15.4)	0.084	6 (23.1)	0.764	4 (15.4)	0.133	3 (11.5)	0.320	4 (15.4)	1.000
non-GCB	34	12 (35.3)		9 (26.5)		11 (32.4)		8 (23.5)		5 (14.7)	

*DLBCL* diffuse large B-cell lymphoma, *GCB* germinal center B cell, *IPI* international prognostic index, *LDH* lactate dehydrogenase.

## Discussion

Given the important involvement of the PI3K/AKT pathway in the pathogenesis of tumors, and given the paucity of datas regarding CNV in PI3K/AKT gene members in DLBCL, we first investigated CNVs using NanoString nCounter’s method [13] in 12 members of the PI3K/AKT signaling pathway in human DLBCL using an nCounter CNV assay. It was found that all PI3K and AKT subunits besides PIK3R1 had CNVs to a different extent, generally, with the frequency ranging from 8.3% to 23%. Among which, CNVs of PIK3CA and PIK3CB were significantly associated with inferior overall survival. Additionally, protein expression of p110α, p110β, p110γ, and p110δ using IHC method were also found to be associated with decreased survival.

CNVs have been found in some genes that are components of the PI3K/AKT pathway in various tumors [14-16]. However, little has been reported about CNVs of the PI3K/AKT in DLBCL. Here we have for the first time detected CNVs in almost all members of the PI3K/AKT signaling pathway in DLBCL. We have revealed CNVs profiles and genetic abnormality as common event in the PI3K/AKT signaling pathway in DLBCL, providing evidence and possible explanation for the pathogenesis of DLBCL on genetic level.

There have been reports showed that PIK3CA gene was happened to CNVs in ovarian cancer [17], cervical cancer [18], and gastric cancer [19]; and that CNVs in PIK3CA were reported to be an independent factor for predicting poor OS of patients with ovarian cancers [17] and gastric cancers [19]. Our result showed that each CNVs of PIK3CA and PIK3CB has significant shorter survival. Either CNVs of PIK3CA or PIK3CB had significant shorter survival too, indicating either PIK3CA or PIK3CB has significant effects on overall survival. Both CNVs of PIK3CA and PIK3CB had the effect trend on survival effect, there was no statistically significance.

In an earlier study, the authors discovered that amplification of PIK3CD was significantly higher in patients who were sensitive to rituximab than that in patients who were resistant to rituximab [20], suggesting that CNVs of PIK3CD may be a useful marker used for chemotherapy sensitivity when selecting the appropriate chemotherapeutic treatment for patients with DLBCL. Alizadeh et al. found that PIK3CG was highly expressed in GCB (germinal center B-cell like), and patients with GCB DLBCL had a significantly better OS than those with ABC (activated B-cell like) DLBCL [21], which was consistent with our detection that PIK3CG expression was significantly higher in GCB cell lines (n = 4).

Among all of the members that we analyzed, the CNVs of PIK3CA and PIK3CB were positively and significantly associated with prognosis compared with other members. Depending on previous research, non-GCB represented poor overall survival than GCB. Although CNVs of PIK3CA and PIK3CB were apparently higher in non-GCB group (80% (8/10) and 67% (8/12), respectively) than that in GCB group, there was no statistically significant. Copy number amplification of either PIK3CA or PIK3CB located in the same region (3q) showed a correlation with poor survival in DLBCL patients, indicating that this region of amplification has significant correlation with survival in DLBCL. There was no significant correlation between the CNV and protein level other than PIK3CA, indicating that CNV of these genes might partially contribute to the aberrant expression of PI3K isoforms supposedly. The inconsistency we discovered here is highly similar to an earlier report by Ye ZQ and colleagues whose finding is that there was inconsistency between CNV and protein differential expression for the most genes [22].

In our present study, not only were CNVs detected in clinical sample of DLBCL, but also it were detected in a panel of six cell lines, including DOHH2, OCI-LY1, OCI-LY8, Toledo, Raji, and Namalwa. From the results obtained at the cell-line level as well as in clinical tissues, it can be seen that CNV was a common event in almost all components of the PI3K/AKT signaling pathway.

Using TMA, we found that of all the members subjected to IHC, p110α, p110β, p110γ, and p110δ protein expression rates were 80%, 81.6%, 81.6%, and 81.6%, respectively. This was similar to the findings of Meadows’s findings in Hodgkin Lymphoma (HL) where they showed that the positive expression of p110α, p110β, p110γ, and p110δ was 97.2%, 29.2%, 54.4% and 80.6%, respectively [23]. The expression of p110α has been reported to be an independent factor for predicting decreased OS for patients with ovarian cancers [17] and gastric cancers [19], although there are no related reports with respect to lymphoma. Protein expressions of p110α, p110β, p110γ, and p110δ showed significant correlation with poor survival.

With regard to AKT, whose active form is phosphorylated AKT (pAKT). It has been reported that p110α amplification was closely associated with pAKT expression. In the study, CNV of PIK3CA was highly associated with aberrant p110α protein expression, which subsequently associated with pAKT, indicating p110α was main isoform for activation of the downstream core protein AKT in DLBCL. PAKT has been extensively reported to be associated with poor prognosis in different types of cancer [15,24-26]. Expression of pAKT has shown a trend towards decreased 5-year survival for patients with DLBCL (*P* = 0.05) [5], while another study showed that high pAKT expression had decreased OS (*P* = 0.036) [27]. In our research, high pAKT expression was associated with poor survival, although statistical significance was not reached. Due to limited number of cases in the study, large cohort study is required to further investigate their relationship and validate our findings.

## Conclusions

In summary, CNVs of PI3K and AKT subunits were a common event in the DLBCL. CNV of PIK3CA is highly associated with aberrant p110α protein expression and subsequent activation of PI3K/AKT pathway. CNVs of PIK3CA and PIK3CB, and aberrant protein expression of p110 isoforms are of great important value for predicting inferior prognosis in DLBCL. Frequent CNVs of PI3K/AKT subunits may play an important role in the tumorigenesis of DLBCL.

## Abbreviations

DLBCL: Diffuse large B-cell lymphoma; GCB: Germinal center B-cell; non-GCG: Non germinal center B-cell; ABC: Active B-cell; IPI: International prognostic index; LDH: Lactate dehydrogenase; TMA: Tissue microarray.

## Competing interests

The authors declared that they have no competing interests.

## Authors’ contributions

WC and YC performed experiments and were responsible for data collection, analysis, interpretation of the results, and writing the manuscript. RB and WW were responsible for conducting the data analysis in cooperation with ZL and PW. MS provided clinical samples for performance of experiments. XZ were responsible for experimental design, analysis and interpretation. All authors have read and approved the final manuscript.
